# Association Between Lower Limb Strength Asymmetry and Gait Asymmetry: Implications for Gait Variability in Stroke Survivors

**DOI:** 10.3390/jcm14020380

**Published:** 2025-01-09

**Authors:** Yungon Lee, Gi Beom Kim, Sunghoon Shin

**Affiliations:** 1 Department of Military Kinesiology, Korea Military Academy, Seoul 01805, Republic of Korea; lyg2311@ynu.ac.kr; 2 Neuromuscular Control Laboratory, Yeungnam University, Gyeongsan-si 38541, Republic of Korea; 3 Department of Orthopedic Surgery, College of Medicine, Yeungnam University, 170 Hyonchung-ro, Daegu 42415, Republic of Korea; donggamgb@hanmail.net; 4 Department of Orthopedic Surgery, Yeungnam University Medical Center, 170 Hyonchung-ro, Daegu 42415, Republic of Korea; 5 Research Institute of Human Ecology, Yeungnam University, Gyeongsan-si 38541, Republic of Korea; 6 School of Kinesiology, College of Human Ecology & Kinesiology, Yeungnam University, 280 Daehak-ro, 221ho, Gyeongsan-si 38541, Republic of Korea

**Keywords:** stroke, gait, lower limb strength asymmetry, gait asymmetry, gait variability

## Abstract

**Background**: Gait disturbances characterized by asymmetries in lower limb strength and gait patterns are frequently observed in stroke patients, which increases gait variability and fall risk. However, the extent to which lower limb strength asymmetry influences gait asymmetry and variability in this population remains unclear. **Methods**: This cross-sectional study included 84 participants, comprising stroke survivors and age- and sex-matched healthy older adults. A portable dynamometer was used to assess lower limb strength, and inertial measurement units to analyze gait parameters. Asymmetry indices were used to quantify strength and gait asymmetries. Statistical analyses included Pearson correlations and stepwise regression to examine the relationships among lower limb strength asymmetry, gait asymmetry, and gait variability. **Results**: Stroke survivors exhibited significantly greater lower limb strength and gait asymmetries than healthy older adults (*p* < 0.001). Knee extension (KE) strength asymmetry was a significant predictor of increased gait variability in stroke survivors (R^2^ = 0.448, *p* < 0.001) but not in healthy controls. Moreover, longer poststroke duration was associated with greater asymmetry in KE strength (r = 0.42, *p* < 0.05) and double support time (r = 0.45, *p* < 0.05). **Conclusions**: Lower limb strength asymmetry, specifically in knee extensors, is a critical determinant of gait asymmetry and variability in stroke survivors. The association between poststroke duration and increased asymmetry indicates the progressive nature of these impairments. These findings emphasize the importance of targeted interventions to reduce strength asymmetry and address chronic impairments in poststroke rehabilitation to improve gait stability and reduce fall risk.

## 1. Introduction

Gait disturbance is one of the major issues faced by stroke survivors [1,2]. Stroke occurs due to vascular impairment in the brain, causing neurological damage that leads to hemiparesis [3]. Consequently, gait patterns become unstable, which is directly explained by the neurological damage [4]. For instance, stroke survivors with hemiparesis exhibit slower gait speed [5], shorter stride length [6], wider step width [7], and increased double support time [8] while walking. These gait characteristics can be considered compensatory strategies aimed at maintaining stability [9], but in the long term, they may exacerbate muscle strength imbalance, increase gait inefficiency, and ultimately, worsen instability. This eventually increases the risk of falls [10]. While previous studies have highlighted the gait impairments caused by hemiparesis, this study specifically examines the direct impact of lower limb strength asymmetry on gait variability and stability in stroke survivors, offering valuable insights into tailored rehabilitation strategies.

Flansbjer, Downham, and Lexell [11] confirmed that the isokinetic knee strength of the hemiparetic lower limb is a significant predictor of gait performance in stroke survivors. Specifically, knee extension (KE) accounted for 42%, 37%, and 50% of the variance in the timed up and go test (TUG), comfortable gait speed, and 6 min walk test (6-MWT), while knee flexion explained 41%, 37%, and 50%, respectively. Knee strength plays a crucial role in determining gait stability and efficiency, while pushing off the ground and lifting the foot [12]. Furthermore, the ankle strength of the hemiparetic lower limb has been identified as an important factor influencing spatiotemporal gait variables, such as stride length and single-leg support time [13]. Weak ankle strength on the hemiparetic side prevents sufficient lifting of the foot off the ground during gait, leading to foot drag, and fails to provide adequate propulsion during foot support. Consequently, this shortens the stride length, slows speed, and reduces gait efficiency [13,14].

In stroke survivors, lower limb strength asymmetry due to hemiparesis significantly challenges gait. Strength asymmetry between both lower limbs is one of the primary factors disrupting gait symmetry [15,16] that occurs as the nonparetic leg compensates for the weaker paretic leg, which undermines balance control and hinders the smooth transfer of the center of gravity [17,18]. Consequently, rhythmic coordination required for muscles and joints to work synergistically in a consistent pattern becomes irregular [19]. Further studies are needed to determine whether such strength asymmetry directly contributes to increased gait variability, a strong fall risk indicator.

Gait variability is a biomechanical indicator sensitive to neuromuscular function and age-related changes [20], making it a powerful predictor of fall risk [21]. This metric was quantified using the SD or coefficient of variation of spatiotemporal gait parameters. Typically, as neuromuscular function deteriorates or aging progresses, the consistency of gait patterns decreases, increasing the gait variability [22]. Elevated gait variability is particularly prominent in patients with major neuromuscular disorders, such as stroke [23], Parkinson’s disease [24], and Alzheimer’s disease [25]. Therefore, gait variability should be effectively managed and consistently monitored for maintaining neuromuscular function and preventing falls.

Research regarding the impact of lower limb strength asymmetry on gait asymmetry in stroke survivors remains limited. Moreover, the extent of the association among these factors is unclear, and the influence of lower limb strength asymmetry on gait variability is poorly understood. Thus, this study aims to determine the relationship between lower limb strength asymmetry and gait asymmetry and to explore how the presence of stroke and strength asymmetry interact to affect gait asymmetry and variability. We hypothesized that asymmetry in lower limb strength would play a key role in causing gait asymmetry and variability. Specifically, it was expected that stroke survivors, who exhibit more pronounced asymmetry, would demonstrate even greater gait variability.

## 2. Materials and Methods

### 2.1. Participants

**Figure 1:**
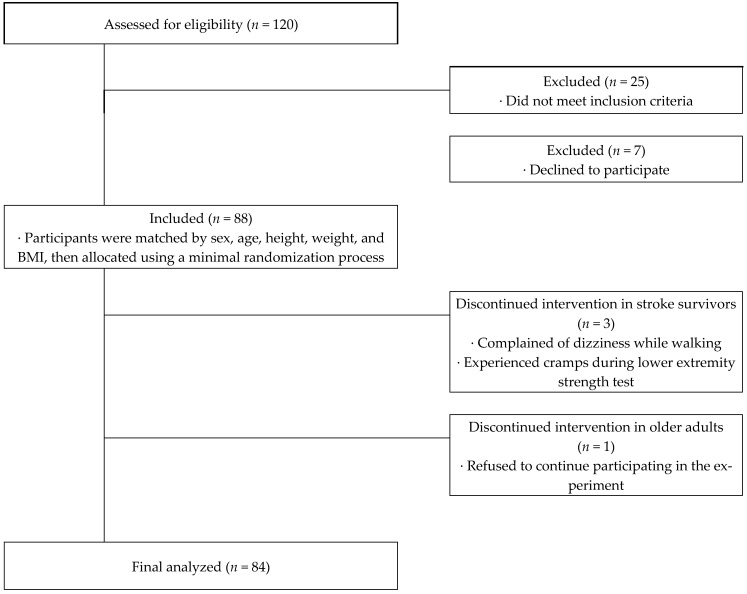
Flow chart showing the experimental design of the study.

This study was designed as a cross-sectional study. A total of 84 male and female participants were included in the experiment and were divided into two groups: stroke survivors (*n* = 30) and a group of healthy older adults (*n* = 54) matched for age, gender, height, and weight (Figure 1). Stroke survivors were recruited from individuals registered at local health centers; whereas, the healthy control group was recruited from local community members. Stroke survivors who were unable to respond to interviews or walk more than 10 m were excluded from the study. Additionally, individuals in the healthy control group with neuromuscular diseases (e.g., Parkinson’s disease, multiple sclerosis, or Alzheimer’s disease) were not eligible to participate.

During the experiment, one stroke survivor reported dizziness while walking and two others experienced muscle cramps, resulting in the termination of their participation. Furthermore, one healthy control participant voluntarily withdrew from the study for personal reasons. All participants signed an Institutional Review Board (IRB) consent form and agreed to participate in the study before the experiment. No adverse effects were reported by the participants after completing the study. This study was conducted with the approval of the university’s IRB. Detailed physical characteristics of the participants are presented in Table 1.

### 2.2. Lower Limb Strength

Lower limb strength measurements were conducted using the MicroFET3 (Hoggan Health Industries, West Jordan, UT, USA). This device is a portable force sensor using a dynamometer’s pressure gauge, capable of measuring the maximum force up to 890 N in Newtons (N) [26]. Previous studies reported that this device has demonstrated high internal reliability for isometric strength measurements [27] and has been widely used in clinical research [28]. The measurement sites included hip flexion (HF), hip extension (HE), hip abduction (HA), knee flexion (KF), KE, ankle plantarflexion (AP), and ankle dorsiflexion (AD).

The measurement protocol for this device was conducted in accordance with the guidelines provided in the microFET3 manufacturer’s user manual and based on the procedures outlined in previous research [26]. Specifically, the strength of both lower limbs was assessed, with each site measured three times. During each trial, participants were instructed to exert maximum force for 3 s, with a rest period of <1 min between trials. The highest value from each site was used for data analysis. The detailed measurement process is as follows: HF was measured by placing the dynamometer on the rectus femoris, while participants, in a seated position, were instructed to push their knees upward with maximum effort. HE was assessed by positioning the dynamometer on the hamstring region, while the participants lay prone and pushed upward with the hamstring. HA was measured by placing the dynamometer at the lateral malleolus, with the participants lying on their sides and lifting their legs upward. KF was measured using the dynamometer fixed at the heel, while participants, in a prone position, pulled their foot inward with maximum force. KE was measured with the dynamometer supported against the dorsum of the foot, as the participants were seated and pushed forward forcefully. For AP, the dynamometer was positioned on the sole, and the participants, lying supine, pressed their ankles downward to exert maximum force. AD was measured using the dynamometer placed on the dorsum of the foot, with participants lying supine and pulling their ankles upward with maximum effort. The collected data were converted into a strength asymmetry index. The formula for calculating the strength asymmetry index is as follows [29,30]:

$$Muscle\;asymmetry\left(\%\right)=\frac{Weak\;side-Strong\;side}{Strong\;side}\times 100$$

### 2.3. Gait Analysis

Gait measurements were conducted using a 7D (3D accelerometer, 3D gyroscope, 1D barometer) inertial measurement unit (IMU; Physilog5^®^, GaitUp™, Lausanne, Switzerland), a sensor that has been validated to demonstrate high validity and reliability [31,32], and its utility has been particularly noted in gait studies involving stroke patients [33]. Participants walked at their preferred speed along a straight course in a gymnasium for 6 min with IMU sensors vertically attached to the dorsum of both feet. The kinematic data collected from the sensors were analyzed at a frequency of 256 Hz.

The analyzed data were categorized into average gait parameters and gait variability. The average gait parameters were presented as mean values and included gait speed (m/s), stride length (m), stride time (s), stance phase (%), swing phase (%), and double support phase (%). The gait variability parameters were calculated as coefficients of variation (CV) [34] and subsequently converted into gait asymmetry indices. The formula for calculating the gait asymmetry index is provided as follows [35]:

$$Gait\;variability,CV\left(\%\right)=\left(\frac{Standard\;deviation}{Mean}\right)\times 100$$

$$Gait\;asymmetry\left(\%\right)=\frac{Weak\;side-Strong\;side}{Strong\;side}\times 100$$

### 2.4. Statistical Analysis

The normality of the data distribution was verified using the Kolmogorov–Smirnov test. Subsequently, independent sample *t*-tests and Mann–Whitney tests were employed to compare the physical characteristics, functional tests, lower limb strength asymmetry, asymmetry of average gait parameters, and gait variability variables between the stroke survivor and healthy control groups. The relationship among stroke onset duration, lower limb strength asymmetry, asymmetry of average gait parameters, and gait variability variables in stroke survivors was analyzed using Pearson correlation analysis.

Before conducting the stepwise regression analysis, potential predictors were identified through Pearson correlation analysis between lower limb strength asymmetry and gait asymmetry or gait variability variables. Independent variables (lower limb strength asymmetry variables) showing significant correlations with dependent variables (gait asymmetry variables and gait variability variables) were selected. Variables such as HF, HE, HA, KF, KE, and AD, identified for gait asymmetry, were included as independent variables in the regression model; whereas, HE, KF, and KE, identified for gait variability, were also included as independent variables in the regression model. All dependent and independent variables were standardized into z-scores for analysis.

To account for group effects, a dummy variable (stroke survivor group, 1; healthy control group, 0) was included as a moderator variable. An interaction term, comprising the control variable independent variable, was generated and incorporated into the regression model. The interaction term was coded as “1” for the stroke survivor group to clarify its effects. This approach distinguished the influence of independent variables on dependent variables between stroke survivors and healthy controls.

When a significant interaction effect emerged as a predictor, separate linear regression analyses were performed for the stroke survivor and healthy control groups. The results were then visualized in a graph. The required sample size for multiple regression analysis was calculated using G*Power 3.1.9.4 software, with a significance level (α) of 0.05, power (1 − β) of 0.90, and effect size (f2) of 0.25. The optimal sample size was estimated to be 81 participants. The effect sizes in the regression model were calculated using Cohen’s f2 and categorized as small (0.02–0.14), medium (0.15–0.34), and large (≥0.35) [36].

## 3. Results

### 3.1. Independent Sample T-Test and Mann–Whitney Test

Table 1 presents the differences in demographic and clinical profiles between stroke survivors and healthy controls. No significant differences were observed in basic demographic variables (age, height, weight, and gender) or NOFY between the two groups. However, stroke survivors took significantly longer in the TUG test compared with the healthy controls (*p* < 0.01), and their RMI mobility scores were significantly lower compared with the healthy controls (*p* < 0.01).

Table 2 shows the differences in lower limb strength, average gait parameters, and gait variability between stroke survivors and healthy controls. Stroke survivors exhibited significantly higher asymmetry in all lower limb strength variables compared with healthy controls (*p* < 0.01). Among the average gait parameters, gait speed was significantly lower in stroke survivors than in healthy controls (*p* < 0.01). Stroke survivors also showed significantly higher values in the stance phase asymmetry, swing phase asymmetry, and double support phase compared with healthy controls (*p* < 0.01). However, no significant differences in stride length asymmetry and stride time asymmetry were observed between the two groups. Regarding gait variability, stroke survivors demonstrated significantly higher values in the stride length CV, stride time CV, stance phase CV, and swing phase CV compared with the healthy controls (*p* < 0.01). Conversely, no significant differences in the double support phase CV were observed between the groups.

### 3.2. Pearson Correlation Analysis

**Figure 2:**
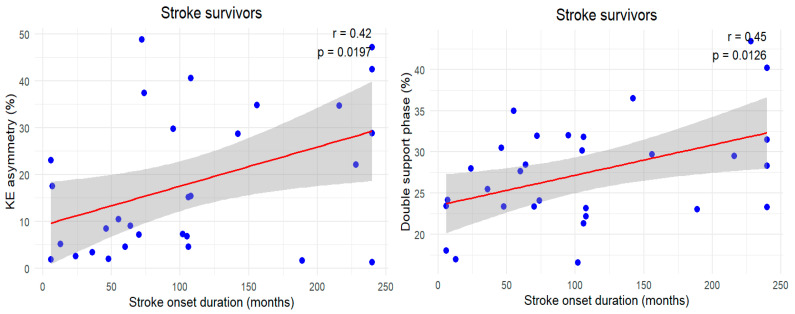
Pearson correlation among stroke onset duration, lower limb strength (asymmetry), average gait parameters (asymmetry), and gait variability in stroke survivors. In stroke patients, when all variables in Table 2 were included in the analysis, the stroke onset duration was significant only for KE asymmetry and the double support phase. Blue dots represent individual data points, the red line represents the linear regression trend line, and the grey area indicates the 95% confidence interval around the trend line.

Figure 2 presents the Pearson correlation analysis results for the relationships between stroke onset duration, lower limb strength asymmetry, asymmetry of average gait parameters, and gait variability variables in stroke survivors. Stroke onset duration was positively correlated with KE asymmetry (r = 0.42) and double support phase asymmetry (r = 0.45) (*p* < 0.05). However, no significant relationships were found between the stroke onset duration and the other lower limb strength asymmetry, average gait parameters asymmetry, or gait variability variables.

**Figure 3:**
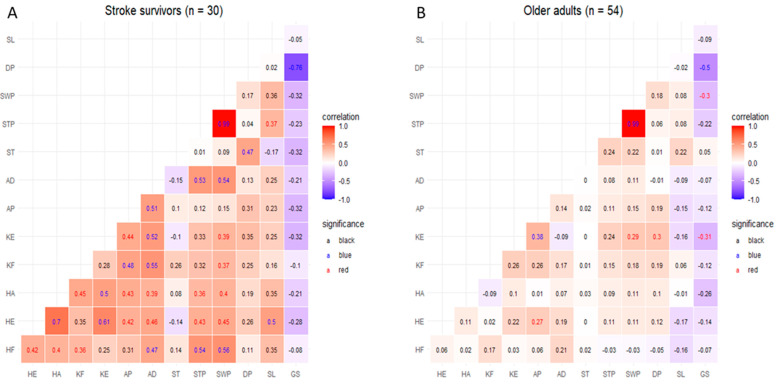
Pearson correlation between the asymmetry of lower limb strength and average gait parameters. Pearson correlation analysis was carried out to identify potential predictors of gait parameters in both stroke survivors and healthy older adults. The results are illustrated in (**A**) for stroke survivors and (**B**) for healthy older adults, with statistical significance highlighted (*p* < 0.01 marked in blue, *p* < 0.05 marked in red, and nonsignificant values in black). The redder the correlation, the closer it is to the positive; whereas, the bluer the correlation, the closer it is to the negative. HF, hip flexion asymmetry; HE, hip extension asymmetry; HA, hip abduction asymmetry; KF, knee flexion asymmetry; KE, knee extension asymmetry; AP, ankle plantarflexion asymmetry; AD, ankle dorsiflexion asymmetry; ST, stride time asymmetry; STP, stance phase asymmetry; SWP, swing phase asymmetry; DP, double support phase; SL, stride length asymmetry; GS, gait speed.

Figure 3 illustrates the Pearson correlation analysis results between lower limb strength asymmetry and average gait parameters in stroke survivors and healthy controls. Among stroke survivors (A), STP was positively correlated with HF (r = 0.54), HE (r = 0.43), HA (r = 0.36), and AD (r = 0.53) (*p* < 0.05). SWP was positively correlated with HF (r = 0.56), HE (r = 0.45), HA (r = 0.40), KF (r = 0.37), KE (r = 0.39), and AD (r = 0.54) (*p* < 0.05). SL was positively correlated with HE (r = 0.50) (*p* < 0.05). In contrast, ST, DP, and GS were not significantly related to lower limb strength asymmetry. In healthy controls (B), SWP and KE were positively correlated with KE (r = 0.29) (*p* < 0.05) and DP (r = 0.30) (*p* < 0.05), respectively; whereas, GS was negatively correlated (r = −0.31) (*p* < 0.05). However, ST, STP, and SL were not significantly correlated with lower limb strength asymmetry.

**Figure 4:**
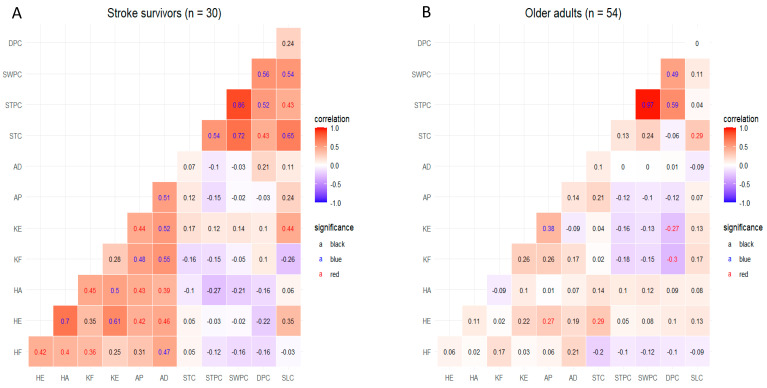
Pearson correlation between lower limb strength asymmetry and gait variability. Pearson correlation analysis was carried out to determine potential predictors of gait parameters in both stroke survivors and healthy older adults. The results are illustrated in (**A**) for stroke survivors and (**B**) for healthy older adults, with statistical significance highlighted (*p* < 0.01 marked in blue, *p* < 0.05 marked in red, and nonsignificant values in black). The redder the correlation, the closer it is to the positive; whereas, the bluer the correlation, the closer it is to the negative. HF, hip flexion asymmetry; HE, hip extension asymmetry; HA, hip abduction asymmetry; KF, knee flexion asymmetry; KE, knee extension asymmetry; AP, ankle plantarflexion asymmetry; AD, ankle dorsiflexion asymmetry; STC, stride time CV; STPC, stance phase CV; SWPC, swing phase CV; DPC, double support phase CV; SLC, stride length CV; CV, coefficient of variation.

Figure 4 presents the Pearson correlation analysis results between lower limb strength asymmetry and gait variability in stroke survivors and healthy controls. Among the stroke survivors (C), SLC was positively correlated with KE (r = 0.44) (*p* < 0.05). However, STC, STPC, SWPC, and DPC were not significantly associated with lower limb strength asymmetry. In healthy controls (D), STC was positively correlated with HE (r = 0.29) (*p* < 0.05); whereas, DPC was negatively correlated with KF (r = −0.30) and KE (r = −0.27) (*p* < 0.05). However, STPC, SWPC, and SLC were not significantly correlated with lower limb strength asymmetry.

### 3.3. Stepwise Regression Analysis

Table 3 outlines the predictors of the average gait parameters. The predictors influencing gait speed were groups and KE (R^2^ = 0.538; *p* < 0.01). Stride length asymmetry was influenced by the interaction variable (groups × HE) (R^2^ = 0.075; *p* < 0.05). Stance phase asymmetry was also predicted by interaction variables (groups × HF, groups × AD) (R^2^ = 0.400; *p* < 0.01). The swing phase asymmetry was influenced by interaction variables (groups × HF, groups × AD) and KE (R^2^ = 0.474; *p* < 0.01). The double support phase predictors were groups and KE (R^2^ = 0.334; *p* < 0.01). No predictors were identified for the stride time asymmetry.

Table 4 presents the predictors of gait variability. The stride length CV predictors were the groups and interaction variables (groups × KE) (R^2^ = 0.448; *p* < 0.01). Stride time CV was predicted by groups (R^2^ = 0.342; *p* < 0.01). No significant predictors were identified for the stance phase CV, swing phase CV, or double support phase CV. 

### 3.4. Post Hoc Analysis for the Interaction Terms

**Figure 5:**
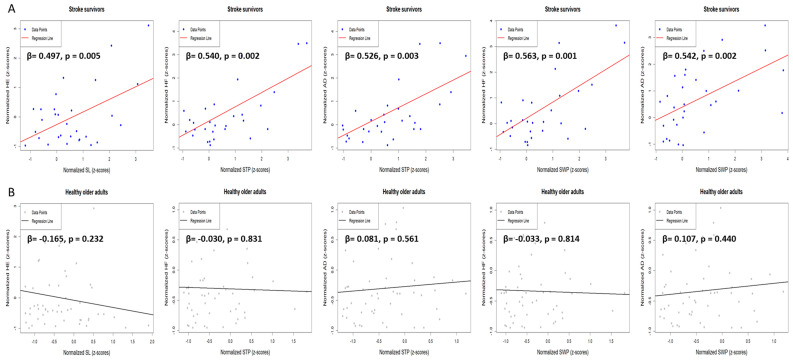
Linear regression analysis results with post hoc tests for the interaction terms. The graph illustrates the linear regression analysis results that indicates the interaction terms between the normalized asymmetry of lower limb strength and gait parameters between the two groups: (**A**) stroke survivors and (**B**) healthy older adults. The regression lines, with the corresponding beta coefficients (β) and *p*-values, reflect the strength and statistical significance of these relationships. HF, hip flexion asymmetry; HE, hip extension asymmetry; AD, ankle dorsiflexion asymmetry; STP, stance phase asymmetry; SWP, swing phase asymmetry; SL, stride length asymmetry.

Figure 5 displays the post hoc results of interaction variables affecting the average gait parameters from the stepwise regression analysis. Among stroke survivors, an increase in HE significantly increased SL (R^2^ = 0.247; *p* < 0.01; f^2^ = 0.32). Additionally, as HF increased, STP also significantly increased (R^2^ = 0.292; *p* < 0.01; f^2^ = 0.41), and a similar increase in STP was observed with increased AD (R^2^ = 0.277; *p* < 0.01; f^2^ = 0.38). Furthermore, SWP significantly increased with higher HF (R^2^ = 0.317; *p* < 0.01; f^2^ = 0.46) and AD (R^2^ = 0.294; *p* < 0.01; f^2^ = 0.41). In contrast, lower limb strength asymmetry did not affect the average gait parameters in healthy controls.

**Figure 6:**
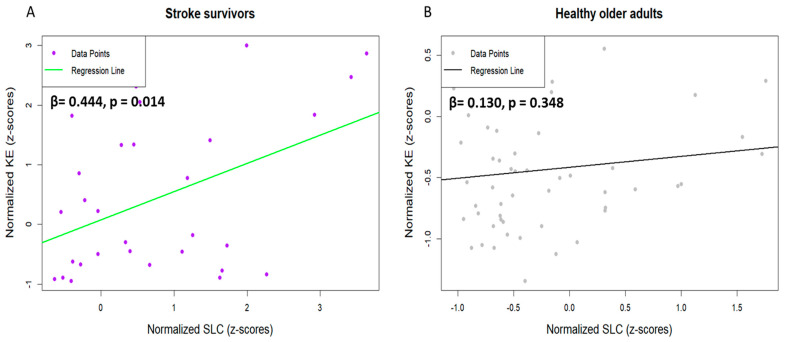
Linear regression analysis results with post hoc tests for interaction terms. The image illustrates the linear regression analysis results examining the interaction terms between normalized lower limb strength asymmetry and gait variability between the two groups: (**A**) stroke survivors and (**B**) healthy older adults. The regression lines, with the corresponding beta coefficients (β) and *p*-values, reflect the strength and statistical significance of these relationships. KE, knee extension asymmetry; SLC, stride length CV; CV, coefficient of variation.

Figure 6 illustrates the post hoc results of the interaction variables influencing gait variability from the stepwise regression analysis. Among stroke survivors, an increase in KE significantly elevated SLC (R^2^ = 0.197; *p* < 0.05; f^2^ = 0.24); whereas, lower limb strength asymmetry did not influence SLC in the healthy controls.

## 4. Discussion

This study investigated the relationship between lower limb strength asymmetry and gait asymmetry and evaluated the interaction between stroke status and lower limb strength asymmetry to influence gait asymmetry and gait variability. We hypothesized that the effects of lower limb strength asymmetry on gait asymmetry and variability would differ between groups and be more pronounced in stroke survivors. The findings revealed that lower limb strength asymmetry was closely associated with gait asymmetry due to stroke, with KE asymmetry being a strong predictor of increased gait variability exclusively in stroke survivors. The detailed results of the study are summarized as follows.

First, stroke patients exhibited lower limb strength, reduced balance ability, gait impairments, and increased gait asymmetry and variability. These results emphasize the direct impact of impaired postural control, muscle weakness, and reduced coordination, all characteristics of stroke patients, on gait performance. Most previous studies support these findings, reporting functional impairments and abnormal gait patterns in stroke patients. Specifically, reduced knee and AD strengths in stroke patients have been associated with increased fall risk [9], gait impairments [17], and gait asymmetry [13,16,17,18,19], which all reflect the physical and functional deficits poststroke [11,14]. These findings emphasize the persistent physical limitations in stroke patients, even after their gait patterns fail to return to normal.

Stroke survivors demonstrated significantly worse performance in gait-related functional assessments, such as TUG and RMI, compared with healthy controls, indicating persistent functional impairments due to stroke. Furthermore, stroke patients exhibited significantly higher asymmetry in all lower limb strength variables compared with healthy controls. Similarly, stroke survivors had higher asymmetry in the stance phase, swing phase, and double support phase compared with the healthy control group. These findings are consistent with those of Lauziere et al. [35], who reported that failed coordination and balance control required for normal gait contribute to gait asymmetry in stroke patients. Furthermore, stroke survivors exhibited significantly higher stride length CV, stride time CV, stance phase CV, and swing phase CV compared with healthy controls. This increased gait variability in stroke survivors likely results from impaired gait control due to damage to the central nervous system.

Balasubramanian et al. [23] reported that increased gait variability while walking in stroke patients is associated with difficulties in balance control. Hausdorff [20] earlier introduced gait variability as an important indicator reflecting complex gait regulation mechanisms rather than simple noise. The magnitude and dynamics of gait variability change in various clinical syndromes, such as falls, frailty, and neurodegenerative diseases. Moon et al. [22] reported that gait variability is significantly elevated in most neurological conditions, including Alzheimer’s disease, amyotrophic lateral sclerosis, cerebellar ataxia, Huntington’s disease, multiple sclerosis, and Parkinson’s disease, compared with healthy controls.

Compared with the control group, stroke survivors exhibited higher asymmetry. This is consistent with the findings of Lauziere, Betschart, Aissaoui, and Nadeau [35], who reported that gait asymmetry in stroke patients occurs from the failure of lower limb coordination and balance control required for normal walking. Furthermore, the stride length CV, stride time CV, stance phase CV, and swing phase CV were significantly higher in stroke survivors than in healthy controls. Such increased gait variability among stroke survivors is likely due to impaired gait control, resulting from damage to the central nervous system.

Balasubramanian, Neptune, and Kautz [23] reported that the increased variability in gait cycles during walking in stroke patients is associated with difficulties in balance control. Hausdorff [20] earlier described gait variability as an important indicator reflecting complex gait regulation mechanisms, rather than mere noise. The magnitude and dynamics of gait variability are reported to change across various clinical syndromes, such as falls, frailty, and neurodegenerative diseases. Moon, Sung, An, Hernandez, and Sosnoff [22] reported a significantly higher gait variability in most neurological disorders, such as Alzheimer’s disease, amyotrophic lateral sclerosis, cerebellar ataxia, Huntington’s disease, multiple sclerosis, and Parkinson’s disease, compared with that in healthy controls.

Second, the relationship between lower limb strength asymmetry and gait asymmetry was particularly evident in stroke patients. Regression analysis revealed that lower limb strength asymmetry explained 24.7–31.7% of the variance in gait asymmetry, indicating that strength imbalance in stroke survivors may reduce gait efficiency and stability. Previous studies have provided evidence that lower limb strength asymmetry significantly affects functional factors and locomotion. For instance, Chun, Kim, Park, and Cho [15] reported that lower limb strength asymmetry in stroke survivors is closely related to functional factors. Specifically, HE, HF, and AD asymmetries were moderate to strongly correlated with the Berg balance scale (r = 0.637, 0.637, 0.494), TUG (r = −0.722, −0.722, −0.689), and the 10 m walk test (r = −0.628, −0.628, −0.631). These findings align with the results of this study, emphasizing the importance of assessing and addressing lower limb strength asymmetry to improve gait ability in stroke patients.

Third, KE asymmetry was identified as the sole significant predictor of increased gait variability in stroke survivors. This result is consistent with previous findings that upper and lower limb strength imbalances negatively impact gait control in stroke patients [15,16]. For example, Chun, Kim, Park, and Cho [15] reported that KE asymmetry is a key predictor of balance and functional gait ability in stroke patients. Specifically, in stroke survivors, unlike in healthy controls, increased KE asymmetry was associated with higher gait variability. This is likely because knee extensor weakness in stroke patients adversely affects the ability to maintain lower limb stability while walking. Thus, rehabilitation strategies for stroke patients should prioritize strengthening KE and reducing asymmetry to enhance gait performance. Conversely, lower limb strength asymmetry did not significantly affect gait function in healthy controls. For instance, Riskowski, Hagedorn, Dufour, Casey, and Hannan [37] reported that, in healthy adults, lower limb strength asymmetry did not significantly influence gait symmetry or variability, indicating that compensatory mechanisms may maintain functional gait even when one limb is weaker in healthy individuals.

Fourth, no lower limb strength asymmetry variables significantly influenced the stride time CV, stance phase CV, swing phase CV, or double support phase CV. This indicates that KE asymmetry alone was a specific factor increasing the stride length CV in stroke survivors. Previous studies have reported that lower limb strength asymmetry is associated with increased gait variability during various physical tasks. For example, Lin, Yang, Cheng, and Wang [13] reported that weakened AD in stroke patients is associated with gait instability. Flansbjer, Downham, and Lexell [11] investigated the relationship between knee strength, gait ability, and perceived social participation. However, this study demonstrated that KE asymmetry was the only variable that significantly predicted gait variability in stroke survivors. This discrepancy may be due to differences in the selection of gait variability indicators, participant characteristics, or experimental settings. In conclusion, this study demonstrates that lower limb strength asymmetry, particularly KE asymmetry, plays a critical role in gait asymmetry and variability in stroke survivors. KE asymmetry was identified as a key factor contributing to gait instability, emphasizing the importance of lower limb strengthening exercises in stroke rehabilitation.

Fifth, this study evaluated the correlations between stroke onset duration and lower limb strength asymmetry, average gait parameter asymmetry, and gait variability variables in stroke survivors. As shown in Figure 2, stroke onset duration was positively correlated with knee extensor strength asymmetry (r = 0.42) and double support phase asymmetry (r = 0.45) (*p* < 0.05). This indicates that, as the time since stroke increases, strength asymmetry in the paretic limb worsens, resulting in compensatory mechanisms, such as prolonged double support time to maintain gait stability. However, no significant correlations were found between the stroke onset duration and other lower limb strength asymmetry, average gait parameter asymmetry, or gait variability variables, indicating the possibility that factors other than stroke onset duration influence gait function. Future research should explore these factors to provide a comprehensive understanding of gait recovery in stroke patients.

Finally, from a practical standpoint in stroke rehabilitation, our findings highlight the importance of prioritizing programs aimed at reducing lower limb strength asymmetry through targeted strength training, particularly for the paretic limb. Specifically, addressing KE asymmetry can be achieved through isokinetic rehabilitation exercises, such as those utilizing an isokinetic dynamometer. These exercises enable the systematic quantification, strengthening, and reinforcement of muscle strength in stroke survivors, as well as effective management and monitoring of their progress [38,39]. Additionally, incorporating balance and gait training can further enhance stability and reduce fall risks in stroke survivors [40,41,42]. These strategies, tailored to the individual’s specific impairments, have the potential to mitigate gait variability, improve overall mobility, and ultimately enhance the quality of life for stroke survivors.

The main limitations of this study, along with several additional considerations, are as follows: First, the sample size in this study was carefully calculated to ensure sufficient statistical power for the analyses conducted, thereby supporting the validity of our conclusions. However, the relatively small sample size, particularly in the stroke survivor group, may limit the generalizability of our findings to broader populations. Future research involving larger and more diverse cohorts, including individuals with varied demographic and clinical characteristics, will likely be necessary to validate these results and expand their applicability. Second, future studies employing a longitudinal design are necessary to better understand the temporal dynamics and causal relationships between lower limb strength asymmetry and gait variability. Such studies could offer deeper insights into how strength asymmetry impacts gait stability over time, ultimately guiding the development of more effective rehabilitation strategies for stroke survivors. Furthermore, longitudinal studies are critical for designing targeted exercise programs aimed at improving gait in stroke survivors and for evaluating their long-term effects on mobility and quality of life. Third, the control group in this study consisted of healthy individuals matched for age, height, weight, gender, and BMI levels to those of stroke survivors, with no reported comorbidities that could affect mobility. This careful selection process was designed to minimize variability and ensure reliable group comparisons. However, the potential influence of unmeasured factors, such as physical activity levels, remains a limitation of this study. Future research should aim to account for these variables to enable a more comprehensive analysis.

## 5. Conclusions

This study evaluated the relationship between lower limb strength asymmetry and gait asymmetry and analyzed the effects of stroke status and lower limb strength asymmetry on gait asymmetry and gait variability. The results demonstrated that lower limb strength asymmetry in stroke patients was closely associated with increased gait asymmetry and variability. Specifically, knee extensor asymmetry was identified as a key factor significantly contributing to increased gait variability. These findings indicate that lower limb strength asymmetry in stroke patients may reduce gait stability and efficiency, while negatively impacting gait control.

Additionally, a longer stroke onset duration was significantly associated with increased asymmetry in the knee extensor strength and double support phase, indicating that as time passes after a stroke, persistent strength weakness and asymmetry may develop, resulting in compensatory mechanisms, such as prolonged double support time to maintain gait stability. Conversely, lower limb strength asymmetry did not have a significant impact on gait symmetry or variability in healthy controls, indicating that compensatory mechanisms allow functional gait to be maintained without significant impairments.

This study emphasizes the importance of strengthening the lower limb muscles, particularly knee extensor asymmetry, as a critical element in rehabilitation strategies for stroke patients to improve gait stability and efficiency. These findings highlight the need for comprehensive rehabilitation approaches focusing on strength training and restoring gait symmetry in stroke patients.

## Figures and Tables

**Table 1:** Demographic and clinical profiles of stroke survivors and healthy older adults.

Parameters	Stroke Survivors (*n* = 30)	Older Adults (*n* = 54)	df	*p*-Value	Effect Size
Age (years)	72.63 (6.75)	73.55 (6.01)	82	0.521	0.15
Height (cm)	163.63 (8.84)	163.39 (7.71)	82	0.898	0.03
Weight (kg)	64.03 (9.16)	65.65 (8.01)	82	0.401	0.19
Gender (males, %)	66.7%	55.6%	62	0.321	0.22
BMI (kg/m^2^)	23.96 (2.47)	24.65 (3.05)	82	0.292	0.24
TUG (s)	15.64 (6.52)	8.50 (1.55)	30	<0.001 ***	1.75
NOFY	0.63 (1.35)	0.14 (0.52)	34	0.068	0.54
RMI	11.96 (1.92)	14.92 (0.26)	29	<0.0001 ***	2.55
Stroke onset duration (months)	106.73 (78.04)	-	-	-	-
Hemiparetic side (right, %)	33.34%	-	-	-	-
The type of stroke (ischemic, %)	80%	-	-	-	-
Use of a walking aid (yes, %)	26.67%	-	-	-	-

Data are mean (SD). BMI, body mass index; TUG, timed up and go test; NOFY, number of falls in the past year; RMI, Rivermead mobility index (max score: 15). Type of stroke: ischemic stroke accounted for 80%, but the remaining cases (20%) were hemorrhagic stroke. Significance set at *** *p* < 0.001. *p*-values and effect size (Cohen’s *d*) show significant differences between stroke survivors and older adults.

**Table 2:** Comparison of lower limb strength and gait parameters between stroke survivors and healthy older adults.

Independent and Dependent Variables	Stroke Survivors (*n* = 30)	Older Adults (*n* = 54)	Total (*n* = 84)	df	*p*-Value	Effect Size
**Lower limb strength**						
Hip flexion asymmetry (%)	22.11 (15.31)	9.66 (8.32)	14.11 (12.73)	38	<0.001 ***	1.05
Hip extension asymmetry (%)	25.63 (15.87)	12.84 (9.56)	17.41 (13.58)	40	<0.001 ***	1.03
Hip abduction asymmetry (%)	21.42 (14.04)	9.64 (8.04)	13.85 (11.93)	39	<0.001 ***	1.07
Knee flexion asymmetry (%)	23.73 (16.69)	12.04 (11.27)	16.21 (14.50)	44	<0.001 ***	0.84
Knee extension asymmetry (%)	18.09 (15.40)	9.68 (8.42)	12.69 (12.02)	38	0.009 **	0.71
Ankle plantarflexion asymmetry (%)	22.39 (16.31)	11.81 (10.06)	15.59 (13.55)	41	0.002 **	0.80
Ankle dorsiflexion asymmetry (%)	29.54 (18.77)	12.95 (9.21)	18.88 (15.53)	36	<0.001 ***	1.19
**Average gait parameters**						
Gait speed (m/s)	0.85 (0.24)	1.28 (0.18)	1.13 (0.29)	82	<0.001 ***	2.05
Stride length asymmetry (%)	1.19 (1.19)	1.19 (1.18)	1.19 (1.18)	82	0.997	0.00
Stride time asymmetry (%)	0.23 (0.29)	0.13 (0.29)	0.16 (0.29)	82	0.128	0.34
Stance phase asymmetry (%)	6.09 (5.49)	2.68 (2.17)	3.90 (4.03)	34	0.003 **	0.89
Swing phase asymmetry (%)	10.42 (9.03)	4.13 (3.33)	6.37 (6.69)	33	<0.001 ***	1.02
Double support phase (%)	27.43 (6.35)	21.41 (4.06)	23.56 (5.75)	42	<0.001 ***	1.16
**Gait variability**						
Stride length CV (%)	6.30 (2.64)	3.60 (1.05)	4.56 (2.20)	82	<0.001 ***	1.46
Stride time CV (%)	4.05 (1.79)	2.26 (0.69)	2.89 (1.47)	82	<0.001 ***	1.44
Stance phase CV (%)	3.46 (1.19)	2.44 (1.03)	2.80(1.19)	82	<0.001 ***	0.92
Swing phase CV (%)	6.09 (2.18)	3.70 (1.56)	4.56 (2.13)	82	<0.001 ***	1.28
Double support phase CV (%)	11.76 (4.87)	10.36 (3.88)	10.86 (4.29)	82	0.151	0.32

Data are mean (SD). Significance set at ** *p* < 0.01; *** *p* < 0.001. The *p*-values and effect size (Cohen’s *d*) are significant differences between stroke survivors and older adults. CV, coefficient of variation.

**Table 3:** Stepwise regression analysis summary of average gait parameters in stroke survivors and healthy older adults (*n* = 84).

Dependent Variables	R^2^	β	Predictors	(95% CI)	VIF	*p*-Value
Gait speed (m/s)	0.538	−0.617	Groups,	(−1.611, −0.949)	1.1	<0.001 ***
		−0.240	KE	(−0.400, −0.080)	1.1	
Stride length asymmetry (%)	0.075	0.273	Groups × HE	(−0.083, −0.646)	1.0	0.012 *
Stride time asymmetry (%)			NA			
Stance phase asymmetry (%)	0.400	0.364	Groups × HF	(0.205, 0.737)	1.4	<0.001 ***
		0.354	Groups × AD	(0.188, 0.711)	1.4	
Swing phase asymmetry (%)	0.474	0.378	Groups × HF	(0.239, 0.741)	1.3	<0.001 ***
		0.273	Groups × AD	(0.076, 0.617)	1.1	
		0.202	KE	(0.015, 0.388)	1.2	
Double support phase (%)	0.334	0.403	Groups,	(0.439, 1.234)	1.1	<0.001 ***
		0.300	KE	(0.109, 0.492)	1.1	

Significance set at * *p* < 0.01; *** *p* < 0.001. Significant interaction terms in the predictors indicated differences in the influence of the independent variables on the dependent variable between the stroke survivor and healthy older adult groups. HF, hip flexion asymmetry; HE, hip extension asymmetry; KE, knee extension asymmetry; AD, ankle dorsiflexion asymmetry; NA, not applicable.

**Table 4:** Stepwise regression analysis summary of gait variability in stroke survivors and healthy older adults (*n* = 84).

Dependent Variables	R^2^	β	Predictors	(95% CI)	VIF	*p*-Value
Stride length CV (%)	0.448	0.500	Groups,	(0.683, 1.392)	1.0	<0.001 ***
		0.328	Groups × KE	(0.199, 0.633)		
Stride time CV (%)	0.342	0.585	Groups	(0.843, 1.583)	1.0	<0.001 ***
Stance phase CV (%)			NA			
Swing phase CV (%)			NA			
Double support phase CV (%)			NA			

Significance is set at *** *p* < 0.001. Significant interaction terms in the predictors indicated differences in the influence of independent variables on the dependent variable between the stroke survivor and healthy older adult groups. KE, knee extension asymmetry; CV, coefficient of variation; NA, not applicable.

## Data Availability

The datasets used in the current study are available from the corresponding author upon reasonable request.
